# Calorie Restriction Decreases Competitive Fitness in *Saccharomyces cerevisiae* Following Heat Stress

**DOI:** 10.3390/microorganisms12091838

**Published:** 2024-09-05

**Authors:** Lucy Hill, Stéphane Guyot, Lucie Bertheau, Hazel Davey

**Affiliations:** 1 Department of Life Sciences, Aberystwyth University, Penglais, Aberystwyth SY23 3DA, UK; 2 Université Bourgogne Franche-Comté, L’Institut Agro, Université de Bourgogne, INRAE, UMR PAM 1517, 21000 Dijon, France; stephane.guyot@agrosupdijon.fr (S.G.);

**Keywords:** *Saccharomyces*, heat stress, calorie restriction

## Abstract

Experiments exposing *Saccharomyces cerevisiae* to glucose limitation (calorie restriction) are widely used to determine impacts on cell health as a model for aging. Using growth on plates and in liquid culture, we demonstrated that calorie restriction reduces fitness in subsequent nutrient-limited environments. Yeast grown in a calorie-restricted environment took longer to emerge from the lag phase, had an extended doubling time and had a lower percentage of culturability. Cells grown under moderate calorie restriction were able to withstand a gradual heat stress in a similar manner to cells grown without calorie restriction but fared less well with a sudden heat shock. Yeast grown under extreme calorie restriction were less fit when exposed to gradual heating or heat shock. Using RNAseq analysis, we provide novel insight into the mechanisms underlying this response, showing that in the absence of calorie restriction, genes whose products are involved in energy metabolism (glycolysis/gluconeogenesis and the citrate cycle) are predominantly overexpressed when yeasts were exposed to gradual heating, whereas this was not the case when they were exposed to shock. We show that both the culture history and the current environment must be considered when assaying physiological responses, and this has wider implications when developing strategies for the propagation, preservation or destruction of microbial cells.

## 1. Introduction

*Saccharomyces cerevisiae*, otherwise known as brewer’s or baker’s yeast, has long been utilised as a model for eukaryotic organisms, particularly in the study of diseases including cancers and neurodegenerative conditions [1]. *S. cerevisiae* has become an important model in studies of aging [2] and particularly for the determination of the impact of calorie-restricted regimes [3,4,5]. This has paralleled an increased interest in the elective reduction of calorie intake to promote health in humans [6,7]. Low-calorie regimens have been shown to extend lifespan and slow the aging process in a wide spectrum of organisms, including invertebrates [8], rodents [9], primates [10] and humans [6]. Whilst these studies are encouraging, the calorie restriction of multicellular eukaryotes can be complex, and experiments using primates require maintenance for several decades. The short generation time and single-celled nature of yeast are highly advantageous in its role as a model organism for these studies.

Goldberg et al. (2009) varied the initial glucose concentration in *S. cerevisiae* cultures (0.2%, 0.5%, 1% and 2% *w*/*v*) and demonstrated that 0.2% glucose resulted in an extension of lifespan of 60% when compared with those receiving 2%. The two major glucose stores for yeast are trehalose and glycogen [11], and yeast cells begin to accumulate trehalose when they enter the post-diauxic phase, with levels continuing to climb throughout this phase and into the stationary phase. This increase in the accumulation of trehalose was observed to be considerably greater in calorie-restricted cells [12] than in those with higher initial glucose concentrations. In contrast, the accumulation of glycogen increased sharply during the logarithmic phase regardless of the initial concentration of glucose within the medium. During the diauxic and post-diauxic phases, calorie-restricted yeast continued to accumulate glycogen, whereas cells grown in the presence of more abundant levels of glucose rapidly consumed it. Although yeast cells grown on 0.5% glucose stored more glycogen by the end of the post-diauxic phase, they consumed it during the stationary phase at a much more rapid rate than those in a medium containing 0.2% glucose [12]. However, whilst studies such as these, which involved a 90% reduction in glucose (calories), are possible in yeast, the majority of eukaryotes would fail to satisfy their basal metabolic rates at this level. Consequently, strategies that involve calorie restriction on some days and eating normally on other days have also gained popularity [13,14].

*S. cerevisiae* is, of course, also an essential organism in biotechnology, where it has long been used in the production of potable alcohol. Within breweries, the initial stresses placed on the yeast cell include high concentrations of sugars but, as the fermentation progresses, these are converted to alcohol. Towards the end of fermentation, nutrient depletion rather than nutrient excess becomes the stressor. Due to the common practice of repitching yeast from a completed fermentation as the inoculum for the next brew, cells are exposed to cyclical nutrient stress (feast and famine). Furthermore, the natural environments of yeast are typically ones of great variability [15], and it is highly likely that nutrient depletions occur periodically in such a setting; therefore, mechanisms are required to allow yeast to maintain viability in a non-proliferating state. In growing cultures, fitness in microbial cells may be defined by readily measurable parameters such as the length of the lag phase and the subsequent doubling time. In a heterogeneous culture, cells with shorter time scales for either of these will have a selective advantage through reaching higher abundance when grown in competition [16].

Despite the above, and whilst changes in the carbon source are known to impact on the lag phase in yeast [17], little work has been carried out on fluctuating nutrient concentrations between sequential cultures. Instead, plentiful supplies of glucose are usually supplied in the laboratory, but this is neither representative of yeast’s natural environment, nor of the many industrial ones in which the nutrient sources rarely remain stable and are in excess for long periods of time. In fruit flies, it has been observed that longevity increases in fluctuating conditions [18]; however, in yeast, it is unclear whether, as well as conferring prolonged lifespan on the cell, calorie restriction also enables cells to develop better tolerance of future nutrient fluctuations. Osmotic stress in *S. cerevisiae* induces activation of the high-osmolarity glycerol (HOG) response, leading to a shift away from glycolysis and to the biosynthesis of trehalose and glycerol. Consequently, we used elevated levels of sodium chloride to determine the impact of calorie restriction on the subsequent ability to grow in the presence of an osmotic challenge. We also investigated the impact of calorie restriction on the fitness of yeast exposed to heat stress. Heat stress has been widely investigated [19,20], and the application of a calorie restriction regime is known to result in the upregulation of heat shock proteins [21,22]. We have previously demonstrated that, in some instances, nutrient-dependent mechanisms are involved in survival from heat stress [20].

Therefore, the aim of the work presented here was to determine whether calorie restriction has an impact on a cell’s fitness in response to changing nutrient levels. We also investigated the influence of calorie restriction on the expression of genes whose products are involved in energy metabolism, with a particular focus on the glycolysis/gluconeogenesis and citrate cycle networks, following exposure to a temperature of 50 °C.

## 2. Materials and Methods

### 2.1. Media Preparation

A standard YPD medium of 1% *w*/*v* yeast extract and 2% *w*/*v* bacteriological peptone was produced; however, the added d-glucose levels were varied across 2% (YPD), 0.5% (YPD-CR) or 0% (YP) *w*/*v*. Additionally, variations of the YPD media containing NaCl were produced with concentrations of 0.25, 0.5 and 0.75 mol·L^−1^ of the added salt. Solidified media were prepared by adding 2% agar. All growth media were autoclaved (121 °C/15 min) before use.

### 2.2. Microorganisms

*S. cerevisiae* yeast (BY4743 *MATa*/*α*; *his3Δ1*/*his3Δ1*; *leu2Δ0*/*leu2Δ0*; *LYS2*/*lys2Δ0*; *met15Δ0*/*MET15*; *ura3Δ0*/*ura3Δ0*) was streaked and grown on YPD agar for 48 h at 30 °C. A single colony was then selected and inoculated aseptically into 20 mL of each of the three growth media (YPD, YPD-CR and YP). Tubes containing the media were incubated at 30 °C for 48 h, with gentle periodic agitation. Each of the tubes was removed from the incubator after 48 h, and serial dilutions in sterile dH_2_O were performed. Cells able to form colonies on each of the solidified media were enumerated using the drop plate method (10 μL) following growth in an incubator at 30 °C for 48 h.

The haploid yeast BY4742 (*MATα*; *his3Δ1*; *leu2Δ0*; *lys2Δ0*; *ura3Δ0*) was used to run the RNAseq analysis. Yeast cells were grown on YPD agar for 48 h at 25 °C. A single colony was then selected and inoculated aseptically into 100 mL YPD liquid medium in a 250 mL conical flask. Flasks containing the media were incubated at 25 °C for 48 h, with gentle periodic agitation. We verified that the behaviour of the BY4742 strain was analogous to that of the BY4743 strain in response to gradual heating and heat shock.

### 2.3. Determination of the Lag Phase’s Duration and Doubling Times

To determine the lag phase and doubling time, cells previously grown on YP, YPD-CR or YPD media were adjusted to the same concentration and inoculated into YPD in a 96-well plate. A Biotek Gen 5 Microplate reader (Biotek Instruments Incorporated, Winooski, VT, USA) was used to incubate the cells at 30 °C for 48 h, taking optical density readings every 20 min. Six replicates of each media type were analysed with 3 µL of each suspension inoculated into 150 μL of YPD. Rows G and H contained the sterile YPD growth medium as controls. Following acquisition of the growth curve data, the 96-well plate was incubated at 30 °C for a further 48 h to ensure no contamination in the uninoculated controls. Three biological replicates of this experiment were performed on different days.

### 2.4. Application of Heat Stress

Cell suspensions grown in the three different glucose concentrations were pelleted by centrifugation at 2500× *g* for 5 min under aseptic conditions. They were adjusted to the same concentration in sterile dH_2_O prior to thorough resuspension. Subsamples (100 µL) were taken and transferred to capped PCR tubes. A BioRad DNA engine (Bio-Rad Laboratories Ltd., Watford, UK) was used to carry out a heat ramp, in which the temperature was increased from 30 to 50 °C at a rate of 0.5 °C·min^−1^ [23], followed by maintenance at 50 °C for a further 30 min. Alternatively, a heat shock protocol was used, which consisted of 1 min at 30 °C, followed by 1 h at 50 °C. Following the heat treatments, serial dilutions were performed and drop plates were prepared for each concentration of glucose and salt. Additionally, conventional plate counts of 100 μL of the undiluted sample were performed, enabling enumeration of samples with low culturable numbers. Samples were also incubated in liquid culture to obtain growth curves as described above.

### 2.5. RNAseq and Transcriptome Data Analysis

Yeast total RNA was extracted using hot acid phenol and quantified using a Nanodrop2100 (ThermoFisher Scientific, Illkirch-Graffenstaden, France). RNA quality was checked by capillary electrophoresis on a Bioanalyzer 2100 (Agilent, Santa Clara, CA, USA). RNA was treated with 1 MBU of Baseline-ZERO^TM^ DNAse (ref#DB0715K, Epicentre, Singapore) for 20 min at 37 °C, followed by a phenol/chloroform and chloroform extraction. RNA was precipitated in 3 volumes of ethanol (96% *v*/*v*) using 0.3 M CH_3_COONa and 15 µg of Glycoblue. After a wash with EtOH (80% *v*/*v*), the pellet was resuspended in 101 µL of RNAse-free H_2_O. After RNA quantification and quality control, 4.5 µg of each RNA (DNAseI-treated) was used for polyA+ isolation using the NEBNext Oligo d(T)25 Magnetic Beads kit (ref#E7490, NEB, Ipswich, MA, USA) following the manufacturer’s instructions. Enrichment of the polyA+ fraction was checked by capillary electrophoresis on a Bioanalyzer 2100. Next, 1 ng of each rRNA-depleted RNA was converted to a library with the Scriptseq V2 RNA-Seq kit (ref#SSV21106, Illumina, San Diego, CA, USA), using the manufacturer’s instructions. After 15 cycles of PCR amplification, the libraries were purified using the Agencourt AMPure XP beads (ref#A63880, Beckman Coulter, Brea, CA, USA) at a ratio of 0.9×.

Library quality was assessed using a high-sensitivity DNA chip on a Bioanalyzer 2100. Library quantification was performed using a fluorometer (Qubit 2.0 fluorometer, Invitrogen, Waltham, MA, USA). Libraries were multiplexed and subjected to high-throughput sequencing using an Illumina HiSeq 1000 instrument with 50 bp single-end read runs and loaded at 12 pM per lane. Quality of the sequencing reads was evaluated by FastQC. Raw sequencing reads were trimmed by Trimmomatic V0.32 with the following parameters: adapters/TruSeq3-SE.fa: 2:30:10; leading: 30; trailing: 30; sliding window: 4:15; MINLEN: 17 and AVGQUAL: 30. Trimmed reads were aligned to the SacCer3 assembly of the *S. cerevisiae* genome using Bowtie2 in sensitive local mode. This generated a *.sam file that was sorted and converted to *.bam, with subsequent removal of non-mapped reads. Visualization was performed in Integrative Genomics Viewer (IGV) (https://igv.org, 27 August 2024) [24].

Differential expression analysis was performed using the DESeq2 package operating in the R environment [25]. Separate analyses were made for all annotated reads, including non-mRNA species, or taking account of only yeast mRNAs. Annotation of the metabolic pathways was performed using the KEGG database (https://www.genome.jp/kegg/pathway.html, 27 August 2024). The processed data are presented in the Supplementary Data File.

### 2.6. Statistical Analysis

Statistical analysis was performed in Minitab version 14. The *p*-values were obtained by performing a one-way unstacked ANOVA, with multiple responses coinciding with the desired pre-culture or treatment.

## 3. Results

### 3.1. Impact of Calorie Restriction on Subsequent Culturability

**Figure 1:**
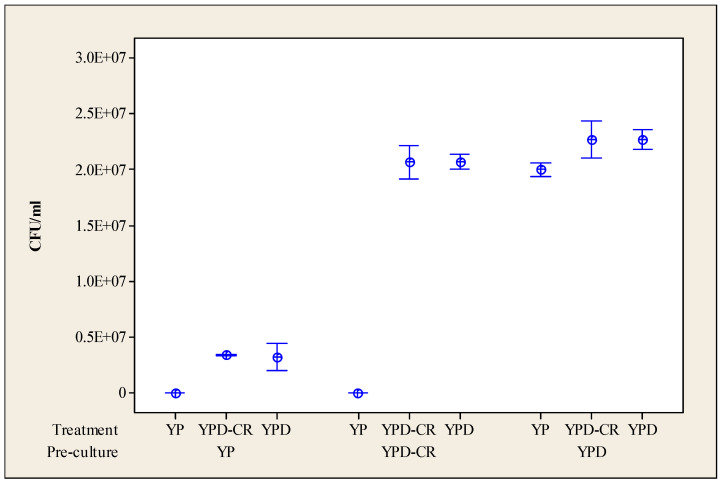
CFUs were counted from agar plates with different concentrations of added glucose. Nine replicates were counted on three separate occasions, resulting in a total of 27 replicates per treatment. The results are divided by the pre-culture and recovery medium treatment groups, with the pre-culture glucose content increasing from left to right along the abscissa. Error bars indicate one standard error from the mean.

Cells were grown in liquid culture containing 0% (YP), 0.5% (YPD-CR) or 2% (YPD) added glucose, as outlined in the Materials and Methods. The cultures were diluted and plated onto solid media containing the three different glucose concentrations, and the number of colonies recovered in each case is shown in Figure 1. Due to the effects of limited glucose on the final cell concentration, it was important to analyse the results relative to YPD agar. Although growth was observed in all three pre-culture tubes, only cells grown on YPD were able to produce colonies on all three treatment plates (different recovery media). There was no observable difference between recovery on YPD compared with YPD-CR for any pre-culture condition, but cells grown on YPD-CR and YP were unable to produce colonies on YP plates. The results indicated that calorie restriction in the pre-culture medium has a negative effect on the cells’ ability to withstand future calorie restriction, but demonstrated that the effects of glucose limitation are reversible in at least a proportion of the population when the cells are subsequently introduced to a medium containing added glucose.

### 3.2. Impact of Calorie Restriction on the Subsequent Lag Phase and Doubling Time

**Figure 2:**
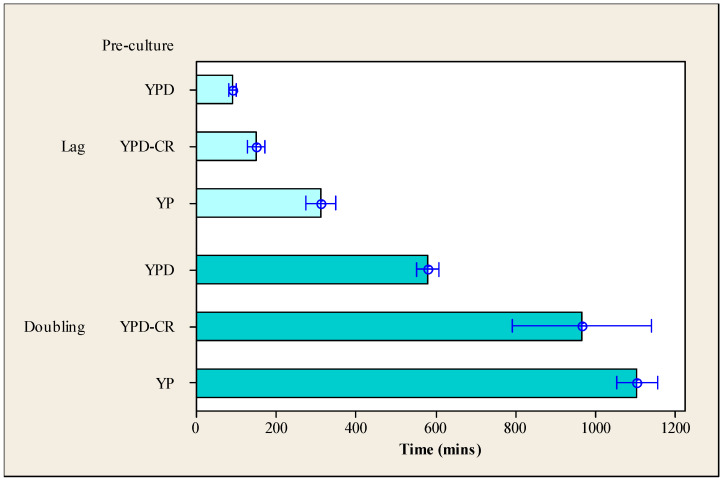
Length of the lag phase and doubling time (in minutes) for cells pre-cultured in one of the three different media prior to inoculation into YPD media. Error bars indicate one standard error from the mean.

Although growth on an agar surface has long been synonymous with estimation of microbial viability [26,27], the method is justly questioned when stressed cells are cultivated [28,29]. All agar plates have a low water activity compared with liquid culture and thus introduce an additional stress, meaning that damaged cells may fail to produce a colony within the incubation time permitted (i.e., culturability provides evidence of viability, but failure to form a colony is insufficient to demonstrate a lack of viability). Consequently, recovery in a liquid medium was also investigated using a 96-well plate reader using YPD as the growth medium (Figure 2).

The mean lag times for cells grown in the three pre-culture conditions and then inoculated into liquid YPD were calculated from a total of 18 replicates obtained over three occasions, as outlined in the Materials and Methods. First, the doubling time was obtained by determining the time taken to reach an optical density twice the value of that measured at the end of the lag phase. The length of the lag phase and the doubling time were calculated and plotted according to the type of pre-culture media (see Figure 2). The results indicated that the higher the glucose concentration in the pre-culture medium, the shorter the lag phase and doubling time when cells were inoculated into YPD. Cells pre-cultured in the YP medium required significantly (*p* < 0.001) longer periods of time to exit the lag phase when compared with those pre-cultured on YPD-CR or YPD media. However, once they had emerged from the lag phase, cells pre-cultured in the YP medium displayed a first doubling time with a length that was not significantly different from that of YPD-CR cells (*p* = 0.455), and this was ~50% longer than the time that cells pre-cultured on YPD required to double the cell number.

### 3.3. Impact of Calorie Restriction on the Heat Stress Response

**Figure 3:**
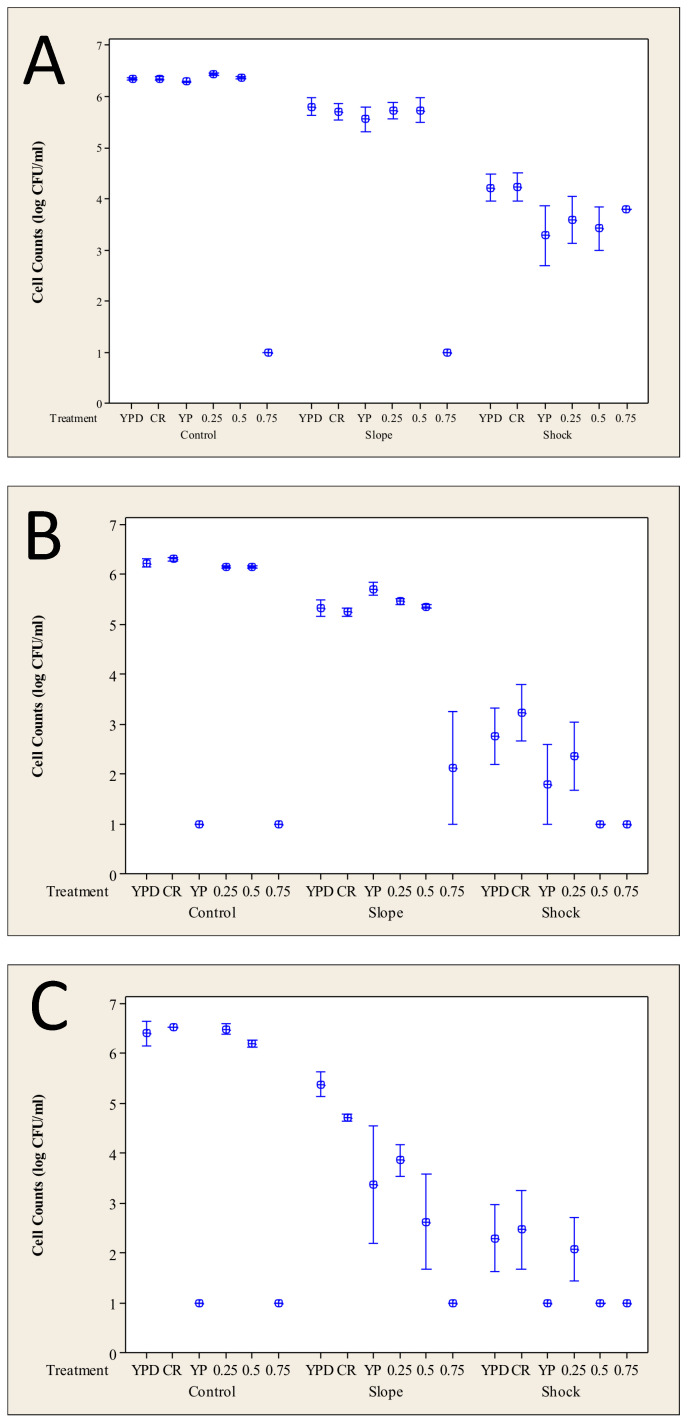
Effect of growth conditions on the response to heat stress. Error bars indicate one standard error from the mean. (**A**) Cells pre-cultured on YPD; (**B**) cells pre-cultured on YPD-CR; (**C**) cells pre-cultured on YP medium. The numbers 0.25–0.75 on the abscissa refer to the concentrations of added NaCl in mol·L^−1^.

Yeast cells grown in the three different pre-culture conditions were subjected to either a rapid or a gradual heat stress, as outlined in the Materials and Methods. Figure 3 shows the impact of the heat stresses on the yeast compared with the unheated controls. Reducing the added glucose from 2% (Figure 3A) to 0.5% (Figure 3B) in the pre-culture medium had little impact on cells exposed to gradual heat stress but led to a greater than a logarithmic reduction in culturability following the heat shock.

Cells that had been subjected to heat stress (and the unheated yeast control) were also challenged with YPD medium to which salt had been added. A proportion of the population that had been pre-cultured on YPD was able to produce colonies after the heat ramp and heat shock on media containing 0.25 or 0.5 mol·L^−1^ sodium chloride. Cells pre-cultured on YPD-CR behaved similarly following gradual heating (heat ramp) but, following the heat shock, were able to produce colonies in the presence of 0.25 mol·L^−1^ sodium chloride but not when this concentration was increased to 0.5 mol·L^−1^. Yeast pre-cultured on YP-medium showed a logarithmic reduction in recovered colonies on 0.25 mol·L^−1^ sodium chloride medium and a reduction of two orders of magnitude with 0.5 mol·L^−1^ sodium chloride following a heat ramp compared with cells pre-cultured on YPD/YPD-CR. Colonies were only rarely produced from YP pre-cultured cells following the heat shock. Interestingly, for yeast pre-cultured on YPD-CR or YP, there was an increase in culturability on YP following the heat ramp compared with the unheated control.

Analysis of the cells pre-cultured on YPD (Figure 3A) media prior to heat treatment indicated that gradual heating had a small impact on the cells’ culturability, whilst a heat shock was seen to have a more detrimental effect, and this is consistent with previous literature [19,23,30]. This pattern was replicated for cells pre-cultured on media with reduced glucose (Figure 3B) or no added glucose (Figure 3C), but the drop in culturability was greatest for cells pre-cultured on the medium without added glucose. Interestingly, whilst cells pre-cultured on YP or YPD-CR media were unable to form visible colonies when placed directly on YP agar, cells exposed to a heat ramp prior to plating produced colonies. In the case of cells pre-cultured on YPD-CR, this was with a similar frequency to cells plated onto YPD or YPD-CR, but for cells pre-cultured on YP, it was with only ~1% of the frequency of colony production on YPD. The heat treatment may result in cell death and the release of nutrients that support cell growth [31,32] via a process referred to as cryptic growth. Alternatively, the additional time in liquid culture prior to plating may have resulted in recovery and repair [33]. These processes are difficult to distinguish.

### 3.4. Impact of Heat Stress on Gene Expression

**Figure 4:**
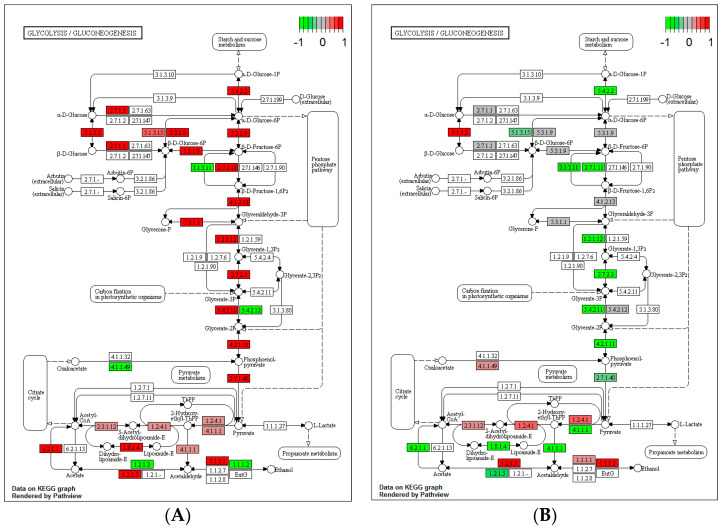
Glycolysis/gluconeogenesis networks of yeast after heat treatment. (**A**) Cells grown on YPD at 25 °C and exposed to a heat ramp on YPD from 25 °C to 50 °C and then maintained at 50 °C for 30 min. (**B**) Cells grown on YPD at 25 °C and exposed to a heat shock on YPD from 25 °C to 50 °C and then maintained at 50 °C for 30 min. The changes are illustrated as log2 fold change values (downregulated genes, green; no change in gene expression, grey; upregulated genes, red). The KEGG website (https://www.genome.jp/dbget-bin/www_bget?sce00010, 27 August 24) provides further information on the genes’ nomenclature. Figures were constructed using the KEGG database (reference sce00010).

**Figure 5:**
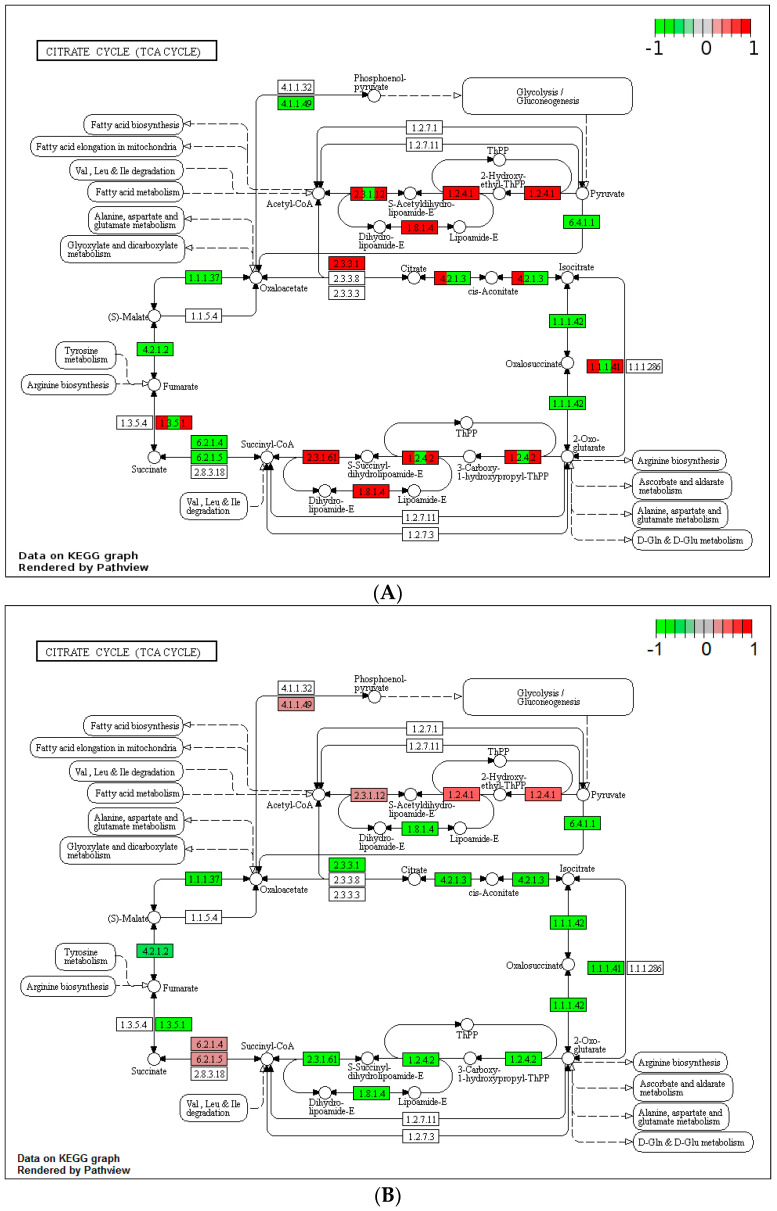
Citrate cycle network of the strain BY4742 after heat treatment. (**A**) Cells grown on YPD at 25 °C and exposed to a heat ramp on YPD from 25 °C to 50 °C and then maintained at 50 °C for 30 min. (**B**) Cells grown on YPD at 25 °C and exposed to a heat shock on YPD from 25 °C to 50 °C and then maintained at 50 °C for 30 min. The fold changes of the genes are illustrated as the values of log2 fold change (downregulated genes, green; no change in gene expression, grey; upregulated genes, red). The reader should refer to the KEGG website (https://www.genome.jp/dbget-bin/www_bget?pathway+sce00020, accessed on 24 April 2019) to know the genes’ nomenclature. Graphs were drawn using the KEGG database (reference sce00020).

Clearly, from the experiments above, the kinetics of heat stress impact on yeast’s culturability. To elucidate the potential mechanisms for this, we investigated changes in the transcriptome occurring during the heat shock and the heat ramp without calorie restriction, as outlined in the Materials and Methods. All samples were found to have a typical RNA profile with an RNA integrity number (RIN) of less than 8.0. Assessment of the library’s quality revealed that all showed a conventional profile without the presence of dimer adapters. The KEGG database was used to analyse the graphs presented in Figure 4 and Figure 5. We focused on the glycolysis/gluconeogenesis and citrate cycle networks, which are, in part, related to glucose catabolism and production of ATP. Generally, after a heat ramp, the genes involved in these networks were upregulated (Figure 4A and Figure 5A), whereas their expression was mainly downregulated after a heat shock (Figure 4B and Figure 5B). Such an observation indicated that in cells grown in the presence of plentiful glucose, heat ramp-induced thermal resistance was related to upregulation of genes directly or indirectly involved in energy production.

Consequently, from the experiments above, we can infer that the mechanism of resistance to a gradual thermal stress is an active process and is dependent upon the prior cultural history of the cells. Growth with restricted glucose concentrations leads to cells that are less fit to survive and grow when changes in glucose concentration or other environmental variations occur.

## 4. Discussion

The behaviour of microbes under constant culture conditions has been widely studied, and the adaptation of yeast to different calorie restriction regimes has been previously investigated. However, here, we set out to determine the impact of changing conditions on fitness to determine the impact of relevant industrial and environmental stresses on yeast’s propagation and survival. We showed that yeast that had been pre-cultured under calorie restriction exhibited slower growth and lower culturability even when moved to richer media. Calorie-restricted yeast also showed an impaired heat shock response, and RNA sequencing analysis revealed altered expression of genes related to energy metabolism in yeast cultures that were cultivated and subjected to heat treatment in the presence of nutrients. More generally, RNA sequencing has shown that when yeast cells were cultured and then subjected to thermal treatment in the presence of nutrients (YPD medium in both cases), the majority of genes whose products play a role in energy metabolism (glycolysis and TCA cycle) were overexpressed following a heat ramp, a response not observed after a heat shock. These results confirmed that in widely used laboratory yeast strains (BY47 collection), the thermal resistance mechanisms induced by a heat ramp are energy-dependent. We have previously demonstrated that the survival of these yeasts during a thermal ramp requires the presence of nutrients, whereas this was not the case for the industrial strain CBS1171 [20]. Other studies have shown that the glycolysis and TCA pathways can also be engaged (increased metabolic flux and/or gene overexpression) when the growth temperature of yeast is slightly elevated to 38–41 °C [34,35]. In light of these observations, it can be reasonably suggested that yeast subjected to a more or less severe caloric restriction struggle to develop thermal resistance mechanisms during the application of a heat ramp, partly due to the lack of overexpression of genes involved in energy metabolism.

Previous studies have indicated that YPD-CR cells accumulate more glycogen than YPD control cells [12], as well as displaying a slowing of their cellular processes to preserve glycogen. Our results confirmed that even after exiting the lag phase on YPD media, inoculums cultured on YPD-CR or YP demonstrated longer doubling times than control cells pre-cultured on YPD, suggesting that cells retain a “memory” of calorie restriction processes and exhibit an economical usage of carbon sources as a result. Previous research has indicated that severely calorie-restricted cells maintained in the cultivation medium for 14 days went on to display doubling times of around 18 days [22], indicating a prolonged adjustment to careful nutrient usage. Although the optical density at inoculation was the same, irrespective of the pre-culture conditions, the final optical density was highest for wells inoculated with yeast that had been pre-cultured on YPD, lowest in wells inoculated with yeast pre-cultured on YP and most variable in wells inoculated with cells pre-cultured in YPD-CR. As the initial optical density readings were observed to be within one decimal place of each other and within the measurable range of the instrument’s specifications, this suggests that cells pre-cultured on YP must expend considerably more energy on maintenance and preparation for growth. However, in contrast to the established concept of adaptation of the whole population during the lag phase following an environmental change [36], more recent work has suggested that observed growth may result from a phenotypically distinct subpopulation [37,38]. The results presented here are consistent with this view, and the longer apparent lag phase may be a consequence of the growth of only a small fraction of the population.

From these results, it is apparent that pre-culturing in YPD-CR media resulted in the detection of high numbers of colony-forming units (CFUs) when subsequently plated onto YPD or YPD-CR agar recovery plates. However, when YPD-CR cells were inoculated into liquid YPD media, they had a doubling time longer than that of cells pre-cultured on YPD media (Figure 2). It is likely that inoculation of cells pre-cultured on YPD-CR into YPD broth resulted in a stalling of cellular processes while the cells adapt to a new environment. This was undetected with solid media, where only the endpoint was recorded.

Following a heat shock, cells reportedly undergo a transient arrest in G1 [39]. Starving cells in G0 have been reported to be significantly more thermotolerant [40] than cells on a nutrient-rich medium. However, this research pre-dates much the more advanced calorie restriction work, and whilst some investigation into yeast’s ability to withstand environmental stresses under a calorie-restricted regime has been performed [41], this is still a relatively unexplored area of research. Whilst *Saccharomyces* yeasts are capable of survival within a broad temperature range, they are typically most stable and capable of proliferation within environments of 30–35 °C [42]. Population growth is possible at temperatures up to 42 °C [43]; however, at temperatures above ~37 °C, the cell membrane’s physiology and carbohydrate flux become altered, resulting in a heat shock response. Above 42 °C, yeast’s RNA polymerase II becomes deactivated, preventing further cell cycling [44], and temperatures above 45 °C are deemed to be within the lethal heat shock range [45]. Nevertheless, all of the values reported above relate to a specific sample of yeast cultured as described in the respective studies; in some cases, careful management of the rate of the increase in temperature can mitigate the impact of heat stress [30]. In the work presented here, a substantial proportion of the yeast cells retained viability and were able to proliferate even in less than optimal conditions following exposure to a temperature of 50 °C.

In conclusion, our results therefore demonstrated the impact of both the pre-culture conditions and heating kinetics on subsequent proliferation, indicating the crucial importance of considering the history and provenance of microbial populations when designing strategies for deactivation and sterilisation. In situations where a prolonged lifespan is desirable, such as yeast used in bottle-conditioned ales, consideration of both genetic and environmental factors during propagation are important [46].

## Data Availability

Data are contained within the article or Supplementary Material. The original data presented in the study are openly available in Gene Expression Omnibus at GSE276309.

## Supplementary Materials

The following supporting information can be downloaded at: https://www.mdpi.com/article/10.3390/microorganisms12091838/s1, Supplementary Data File: Supp data 1_RNAseq_KEGG_SG.csv.
